# Lower glutamate and GABA levels in auditory cortex of tinnitus patients: a 2D-JPRESS MR spectroscopy study

**DOI:** 10.1038/s41598-022-07835-8

**Published:** 2022-03-08

**Authors:** B. Isler, N. von Burg, T. Kleinjung, M. Meyer, P. Stämpfli, N. Zölch, P. Neff

**Affiliations:** 1 Department of Otorhinolaryngology, University Hospital Zurich, (USZ), University of Zurich (UZH), Zurich, Switzerland; 2 Faculty of Medicine, University of Zurich (UZH), Zurich, Switzerland; 3 Division of Neuropsychology, University of Zurich (UZH), Zurich, Switzerland; 4 University Research Priority Program ‘Dynamics of Healthy Aging’, University of Zurich (UZH), Zurich, Switzerland; 5 Department of Psychiatry, Psychotherapy and Psychosomatics, Psychiatric University Hospital Zurich, University of Zurich (UZH), Zurich, Switzerland; 6 Institute of Forensic Medicine, University of Zurich (UZH), Zurich, Switzerland; 7 Department of Psychology, Center for Cognitive Neuroscience, University of Salzburg, Salzburg, Austria; 8 Department of Psychiatry and Psychotherapy, University of Regensburg, Regensburg, Germany; 9 Institute of Bioengineering, Center for Neuroprosthetics, École Polytechnique Fédérale de Lausanne, Geneva, Switzerland; 10 Department of Radiology and Medical Informatics, University of Geneva, Geneva, Switzerland

**Keywords:** Auditory system, Cortex, Neurophysiology

## Abstract

We performed magnetic resonance spectroscopy (MRS) on healthy individuals with tinnitus and no hearing loss (*n* = 16) vs. a matched control group (*n* = 17) to further elucidate the role of excitatory and inhibitory neurotransmitters in tinnitus. Two-dimensional J-resolved spectroscopy (2D-JPRESS) was applied to disentangle Glutamate (Glu) from Glutamine and to estimate GABA levels in two bilateral voxels in the primary auditory cortex. Results indicated a lower Glu concentration (large effect) in right auditory cortex and lower GABA concentration (medium effect) in the left auditory cortex of the tinnitus group. Within the tinnitus group, Glu levels positively correlated with tinnitus loudness measures. While the GABA difference between groups is in line with former findings and theories about a dysfunctional auditory inhibition system in tinnitus, the novel finding of reduced Glu levels came as a surprise and is discussed in the context of a putative framework of inhibitory mechanisms related to Glu throughout the auditory pathway. Longitudinal or interventional studies could shed more light on interactions and causality of Glu and GABA in tinnitus neurochemistry.

## Introduction

Tinnitus is a condition in which the affected individuals experience a persistent sound in one or both ears, or in the head. Recently, this definition has been expanded to include a differentiation between the auditory phantom perception and a (related) disorder, characterized by high levels of distress^1^. The tinnitus perception is usually described as a high-pitched tone, hiss, noise, ringing, or a combination thereof. About 15% of all adults suffer from chronic subjective tinnitus^2,3^, whereas about 50% of all adults report experiencing tinnitus-like phantom sounds in an anechoic environment^4,5^.

The etiology and pathophysiology of this disabling condition is widely unresolved. Although most individuals with hearing loss do not develop tinnitus, most individuals with tinnitus have an abnormal audiogram, suggesting that the tinnitus symptoms (at least, initially) originate in the cochlea. While tinnitus mostly originates in the periphery, mechanisms of the central auditory pathway and of the auditory cortex are critical in its generation, maintenance, and persistence. The reduced auditory input from the cochlea by initial peripheral deafferentation and consequently reduced firing rates in the cochlear nerve may cause increased spontaneous activity in the auditory pathway^6,7^. The reduced input to the central neurons is theorized to lead to an increased response to the remaining incoming signals. This imbalance, in which a given input generates a stronger output or vice versa, is referred to here as “gain” and can also be interpreted as reduced inhibition^8^. Gain control is of paramount importance to maintain homeostasis and to control the functioning of all physiological processes. The neural changes including neuronal excitability, receptor expression or the production and release of neurotransmitters regulating the balance are subsumed under the concept of homeostatic plasticity^9^. Alternatively, predictive brain frameworks interpret changes in the cortical homeostatic plasticity in response to the central gain to be part of the pathogenesis of tinnitus^7,8^. The role of these activity increases in the form of neuronal hyperactivity, bursting discharges and increased cortical neural synchrony in tinnitus pathogenesis is still unresolved but might be a result of lack of inhibition in the central auditory pathway^10,11^.

A main focus of the loss-of-inhibition hypothesis in tinnitus is the role of $\gamma$-amino butyric acid (GABA), an inhibitory neurotransmitter, which, among others, is responsible for maintaining the balance between excitation and inhibition. Furthermore, GABA is found at many levels of the auditory pathway. Insufficient inhibition due to lower concentration, altered distribution in the tissue, or changed receptor density or affinity of GABA could thus be a cornerstone in the development of tinnitus. GABA has been associated with various physiological and pathophysiological neurological processes. In the central auditory system, GABA has been shown to be the main inhibitory neurotransmitter^12^. Based on earlier findings in animals, Gao and colleagues^13^ established a relation between the pure tone averages in presbycusis and reduced GABA concentration in the auditory regions. In animal studies, systemically administered GABA agonists eliminated psycho-physical evidence of tinnitus in rats for a short period of time^14^, a finding which could be transferred to human tinnitus perception. With improvements of magnetic resonance spectroscopy (MRS), it became possible to measure GABA concentrations with MRI scanners in vivo. In an MRS study with rats with induced tinnitus, decreased GABA levels in the medial geniculate body, a relay for ascending auditory information, could be observed^15^. In a human MRS study, a GABA deficit in the right auditory cortex was found in individuals suffering from tinnitus compared to healthy individuals^16^.

Based on the same loss-of-inhibition theory, an up-regulation of the excitatory neurotransmitter Glutamate (Glu) is suspected to play a role in disrupting the excitation/inhibition balance^11^. Research has been able to demonstrate similar molecular changes along the entire auditory pathway to the auditory cortex: In the above-mentioned MRS study, Glu was elevated in the dorsal cochlear nucleus and in the primary auditory cortex (i.e., A1)^15^. The cochlear nucleus (CN) receives somatosensory and auditory nerve projections which are both glutamatergic with a different subtype of Glu receptors^17^. Cochlear damage results in a redistribution of these receptor subtypes in the CN, where an up-regulation of non-auditory Glu receptors (VGLUT2) in the CN could be observed^18^. This increased glutamatergic input could be responsible for increased spontaneous firing rates in the CN neurons, which is proposed as a correlate of the tinnitus sensation. The application of low frequency, repetitive transcranial magnetic stimulation initiates inhibitory cortical mechanisms, resulting in a down-regulation of Glu in the auditory cortex of the left hemisphere and a significant reduction in tinnitus loudness levels^19^. In unilateral tinnitus, lower levels of Glu were observed in the hemisphere ipsilateral to the tinnitus ear compared to the other hemisphere^20^. Notably, however, these results were presented at a conference; a peer-reviewed publication is still outstanding. Miyakawa and colleagues^21^ induced tinnitus in a knockout mouse model through the inhibition of Glutamate decarboxylase 65 (GAD65) expression. This enzyme catalyzes the decarboxylation of Glu to GABA^22^. Changes in the functioning of this enzyme could affect the concentration of Glu and GABA, as well as the sensitivity of the respective receptors. While many studies indicate a shift in the balance of excitation/inhibition towards excitation in the auditory system of individuals with tinnitus, a consistent theory of the influences of the various neurotransmitters does not yet exist.

Proton Magnetic Resonance Spectroscopy (^1^H-MRS) allows for the non-invasive detection and quantification of metabolites within a predefined region of interest. The signals obtained provide a spectrum with a limited number of identifiable peaks, representing different molecules. Using a 3T scanner, current techniques are able to measure around 18 metabolites in the human brain^23^. Due to the limited chemical shift dispersion, peaks from different metabolites overlap and metabolites with low concentrations might remain hidden in the spectrum. There are several strategies to address this problem of overlapping metabolite signals. For example, measurements at field strengths higher than 3 Tesla can help to separate overlapping signals. So-called editing sequences are used to select specific metabolites such as GABA or gluthatione^24^, with the disadvantage that information about the other metabolites is lost or limited. Another possibility for improvement is offered by two-dimensional MR spectroscopy. Here, the available information is spread over two dimensions to reduce the signal overlap. Two-dimensional J-resolved spectroscopy (2D-JPRESS) is one of the available methods for 2D MRS^25^. This sequence exploits the J-coupling, a through-bond interaction between proton spins, which is observed for metabolites like GABA, Glu and Glutamine, Lactate and many others. 2D-JPRESS builds on the conventional single-voxel MRS protocol PRESS, where it additionally encodes the J-coupling evolution in a second dimension by varying the acquisition echo time. By changing the echo time, only the signals from J-coupled protons collect a phase which alters the final 2D spectrum in that the corresponding peaks are shifted into the second dimension. In consequence, 2D-JPRESS improves for the resolution of overlapping signals such as GABA, Glu and Glutamine, which are critical for the planned analyses here^23^. It has been shown that 2D-JPRESS may be the most accurate sequence for detecting GABA despite the disadvantage of a longer acquisition time and thus the potential for more movement artifacts^26^. In addition, it has been demonstrated that editing sequences, like MEGA-PRESS (MEscherGArwood Point RESolved Spectroscopy), tend to overestimate GABA due to scaling factors, limited flexibility in the baseline definition and macromolecular contamination^26,27^. Taken together, these technological advantages qualify 2D-JPRESS to be the most suitable MRS method at 3 T to resolve Glu and Glutamine and to quantify GABA.

Cacace and colleagues^28^ suggested MRS as a non-invasive analysis technique to further analyze metabolic and neurochemical biomarkers in the central nervous system associated with tinnitus in humans. Since its development, MRS has become an meaningful tool in the study of brain function in both healthy and pathological states. Recently, MRS has been used to investigate age-related changes in presbycusis^29^. The major etiological driver of presbycusis is the loss of hair cells in the inner ear. MRS demonstrated metabolic differences in the auditory cortex and showed a decline in Glu and n-acetylaspartate levels in higher age, as well as increased lactate levels in pronounced presbycusis^29^. Using the same method, another study found reduced GABA concentrations in individuals suffering from presbycusis, including correlations between the pure tone audiogram and GABA concentration in the whole group^13^. To the best of our knowledge, only two MRS studies of tinnitus, in which N-acetylaspartate, GABA, choline and creatine were measured^16,30^, have been published so far.

For the first time, we analyzed metabolites in the auditory cortex of individuals with tinnitus using the 2D-JPRESS method. The discussed advantages of 2D-JPRESS should allow for a feasible estimate of GABA concentrations as well as a better resolution of the Glu-Glutamine peaks in order to quantify Glu in auditory cortex.

We expected to find lower GABA and higher Glu concentrations in the primary auditory cortex in tinnitus patients compared to healthy adults controlling for hearing loss. Furthermore, we assumed that such findings would be corroborated by additional correlations of tinnitus perceptual parameters, such as tinnitus loudness, minimum masking level (MML), and residual inhibition (RI).

## Methods

### Participants

Twenty adults with tinnitus were included and matched with 20 healthy adults acting as the control group. Participants with tinnitus were recruited from clinical consultations at the University Hospital Zurich and by accessing a participant pool shared with the University of Zurich. The control group was recruited at the university hospital and the university campus with flyers, in clinical practice, or from institutional staff. Groups were matched with respect to gender, age, and hearing loss (case-control matching). Absence of clinically relevant hearing loss (both groups) as well as chronic subjective tinnitus with a minimal duration of 6 months (tinnitus group) were defined as inclusion criteria. Critical hearing loss was defined as pure tone average $\ge$ 25 dB HL between 0.5 and 4 kHz and $\ge$ 35 dB between 4 and 8 kHz. Further, all participants were right-handed and had to meet all safety criteria for MRI. Exclusion criteria were vestibular, inner ear or other otological diseases, neurological or psychiatric disorders, professional musicians defined by more than 6 h of practice per week, regular intake of drugs or drug intake in the week before magnetic resonance imaging. The study was approved by the local ethics committee “Kantonale Ethikkommission Zürich” (KEK-ZH, approval number 2019-00479) ) and was carried out in accordance with the Declaration of Helsinki. All participants were thoroughly informed about the study before signing an informed consent. Written informed consent was obtained from all participants.

### Screening and psychometry

For screening purposes, participants had to provide information on regular medication use, head surgery, neurological or psychiatric illnesses, other illnesses, use of alcohol, tobacco or other drugs, and information regarding illnesses concerning the ear or auditory pathway (deafness, use of hearing aid, inner ear diseases and hyperacusis) in particular. Participants were furthermore screened to exclude cases of pregnancy, claustrophobia, body implants (e.g., dental implant, pacemaker), tattoos, piercings, and movement risks for the duration of the MRI (e.g., through susceptibility to pain or cold). The ability to remain still is a critical consideration in the counteraction of movement artifacts in MRI spectroscopy with its long scanning times (up to 24 min per ROI, in this study). Since right-handedness was an inclusion criterion, participants also had to complete a handedness questionnaire (Edinburgh Handedness Inventory $=$ EHI)^31^.

To assess demographic data, medical history, tinnitus characteristics (tinnitus group), psychological well-being and quality of life, the participants had to fill in an online questionnaire^32^. Participants then had to complete questionnaires on quality of life (WHO-Quality of Life-BREF)^33^, stress (Perceived Stress Questionnaire 20 = PSQ-20)^34,35^, depression (Beck Depression Inventory = BDI)^36^, anxiety (Beck Anxiety Inventory = BAI)^37^ and personality (Big Five Inventory = BFI-2)^38^. In the tinnitus group, the Tinnitus Handicap Inventory (THI)^39,40^ was assessed. In order to gain additional information about potential hyperacusis, participants had to complete a German hyperacusis questionnaire (Geräuscheüberempfindlichkeits-Fragebogen = GUEF)^41^.

Lastly, tinnitus participants had to indicate the current loudness, burden and ignorability of their tinnitus on a visual analogue scale (VAS).

### Audiometry and tinnitometry

#### Pure tone thresholds

The entire audiological and tinnitometric assessment was performed in a sound-attenuated, double-walled booth with circumaural headphones (Sennheiser HD 280 for frequencies from 250 to 8000 Hz and Sennheiser HD 200 for frequencies from 8000 to 14000 Hz; Sennheiser, Old Lyme, USA) on a certified and calibrated audiometric system (Interacoustics, Middelfart, Denmark). Pure tone thresholds were assessed to quantify peripheral hearing and loudness discomfort levels (LDL). Normal hearing was defined as a pure tone average at or below 25 dB hearing loss for frequencies of 500 to 4000 Hz and at or below 35 dB hearing loss for frequencies of 4000 to 8000 Hz. Participants who did not meet this criterion were excluded. Thresholds were measured using a probe-detection paradigm with pure tones presented for 250 ms at frequencies of 250, 500, 1000, 2000, 4000, 8000, 9000, 10,000, 11,200, 12,500 and 14,000 Hz for each ear.

To determine the LDL, the volume was set to 75 dB at 1000 Hz and then continuously raised in 5 dB steps (every 5 s) until the subject gave the signal that this volume was uncomfortable. When the volume exceeded 110 dB the test was stopped to prevent any hearing damage.

Audiometry and tinnitometry was completed before the MRI scan, which was performed on a different site, and with a few exceptions on a different day or at least with a break of 2 h.

#### Tinnitometry

Tinnitus pitch, loudness, minimal masking level (MML), and residual inhibition (RI)^42^ were assessed with the audiometric equipment at the university hospital, described in detail below. The tinnitus patients were asked to define their tinnitus character by means of intensity and frequency. Tinnitus patients were presented with the pure tones from the set of the pure tone average frequencies on the affected ear(s) and had to choose the frequency most similar to their tinnitus perception. Following that, the frequency was further adjusted in steps of 5 Hz to fine-tune the matching. When the frequency was matched, we determined the loudness in steps of 5 dB until the presented stimulus matched the subjective tinnitus loudness of the participant.

MML was measured by presenting the participant broadband noise in increasing intensity of 5 dB steps until the tinnitus perception was masked by the white noise. To probe RI, participants were presented with white noise 15 dB above the MML level for 30 s and had to rate tinnitus loudness after white noise stimulation offset on a scale from $-5$ to 2 ($-5 =$ full suppression, 0 $=$ no change, 2 $=$ tinnitus significantly louder).

### MRI

MRI data acquisition for both the structural and MRS measurements was performed on a 3 T whole-body MR scanner (Achieva with dStream Upgrade, Software Release 5.4.1, Philips Healthcare, Best, the Netherlands), equipped with 80 mT/m gradients and a 32-channel receive head coil. All participants were scanned at random times between 07.00 a.m. and 11.00 p.m. on all days of the week with no differences between groups (t-test, *p* $$= $$0.36).

#### Structural

The anatomical sequence applied was a high resolution T1-weighted anatomical 3D Turbo-Field-Echo (TFE) sequence with echo time (TE) $=$ 3.79 ms, repetition time (TR) $=$ 8.18 ms, acquisition matrix $=$ 256 $\times$ 256, 160 slices per volume, field of view (FOV) $=$ 240 $\times$ 160 $\times$ 240 mm, flip angle ($\alpha$) = $90^\circ$ and isotropic voxel size $=$ 0.94 $\times$ 0.94 $\times$ 1 mm.

For the cortical surface reconstruction, we used the FreeSurfer image analysis suite (version 6.0.0.). The FreeSurfer software bundle is freely available and documented online (http://freesurfer.net). The software generates individual, high precision cortical reconstructions which are then projected onto surface models. Its pipeline automates several processing steps of MRI preprocessing, transformation, normalization, registration, segmentation of different brain tissues, tesselation, inflation, and finally the extraction of differential anatomical measures from the reconstructed surfaces^43,44^. Cortical thickness, surface area, and volume are calculated as the main measures of interest at each vertex of the surface. Cortical thickness has been validated by using manual segmentations^45^ or histological analysis^46^. In addition, this method is an established generator of reliable results in healthy adults in the absence of any operator intervention in the automated pipeline^47^.

In our study, structural MRI was assessed to control for potential structural confounders (i.e., differences between groups) of the MRS analysis. A whole-brain analysis was performed checking for differences in cortical thickness, surface area, and volume between groups controlling for total intracranial volume. This analysis was performed with QDEC, a FreeSurfer tool for whole-brain statistical modeling, for each hemisphere and corrected for multiple comparisons on the vertex level using the built-in Monte Carlo simulation with 10,000 permutations and an initially clusterforming threshold of *p* $\le$ 0.05. An additional post hoc Bonferroni correction was applied to correct for the hemisphere-wise analyses.

#### Spectroscopy

**Figure 1:**
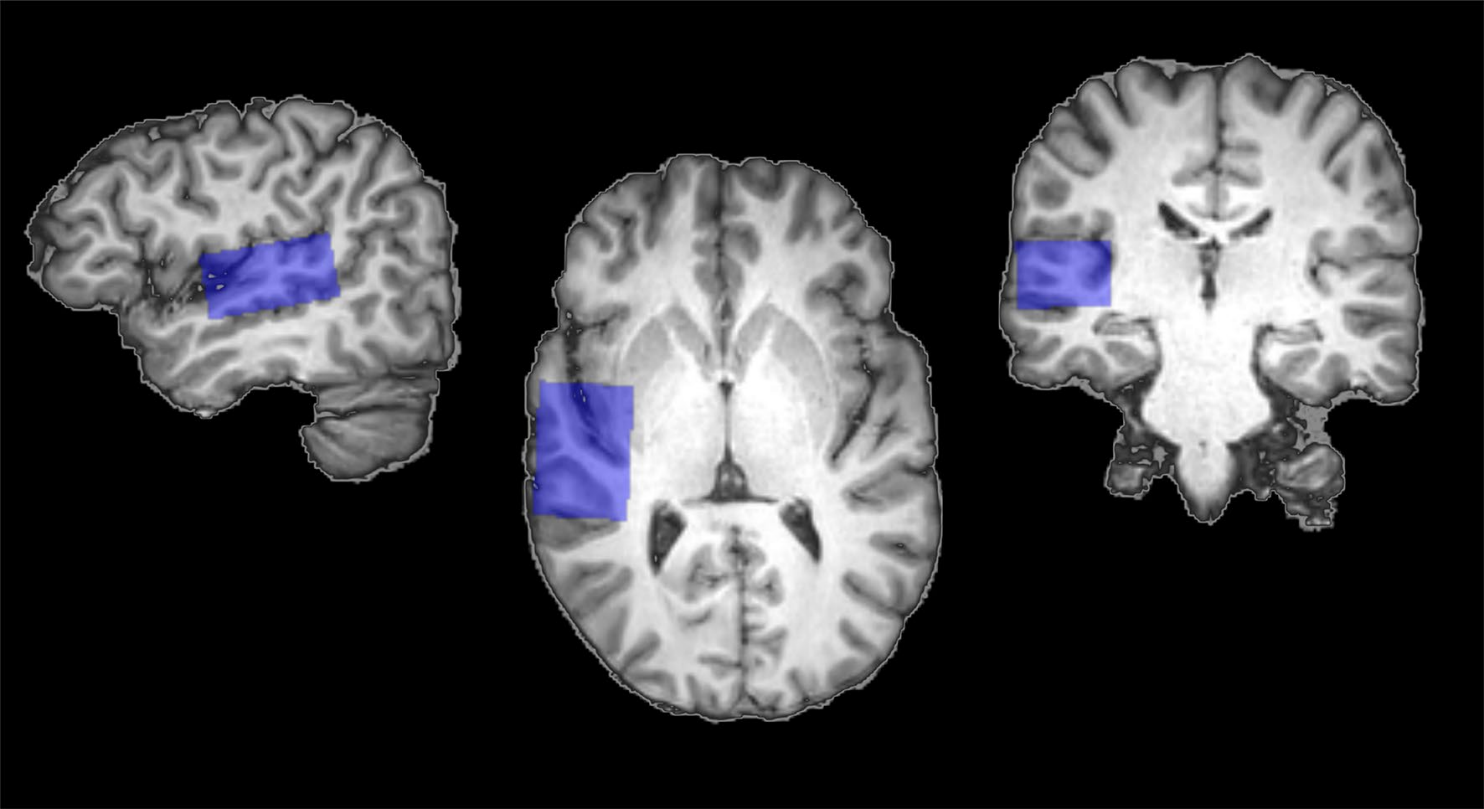
MRS ROI voxel. Right voxel (radiological convention) colored in blue and in sagittal, horizontal, and coronal view (from left to right). The voxel covers Heschl’s Gyrus, parts of superior temporal gyrus, and insular cortex. No differences in voxel size or composition between the two hemispheres were found.

A 2D-JPRESS sequence was used for the MRS acquisition^25^. A nominal voxel size of 38 mm (L-R) $\times$ 26 mm (I-S) $\times$ 46 mm (A-P) (45.5 cm^3^) was analyzed using 2D-JPRESS with a minimal echo time (TE) of 30 ms^48^. TE was increased in steps of 2 ms with 100 steps in total. $\Delta$TE sampling for each step started $\Delta$ TE/2 earlier with respect to the echo-top^25^. We chose a repetition time (TR) of 1600 ms and eight averages were acquired for each TE step. Water suppression was achieved via the VAPOR method^49^. For each TE a non-water suppressed spectrum was acquired which was used for eddy current correction and as water reference signal for metabolite quantification. The total scan time accumulated to 24 min per segment (i.e., voxel) and the right segment was always scanned first in all participants. To reduce chemical shift displacement effects and render the data collection consistent for all metabolites in the range from 1.3 to 4.0 ppm, selective saturation pulses were used (OVERPRESS)^50^. With this the effective voxel size reduced to 28.4 mm (L-R) $\times$ 19.4 mm (I-S) $\times$ 46 mm (A-P) (39.3 cm^3^). Figure 1 illustrates the effective voxel position and extent in an example individual T1-weighted brain MRI. The spectral bandwidth was 2000 Hz with a number of 1024 points per spectrum. Additionally we used an automated B0 shimming routine. To be able to correct for the relaxation attenuation of the water signal obtained within 2D-JPRESS, we acquired an additional non-water suppressed 1D spectrum with a long repetition time of 10 s and a series of 6 echo times (30, 63, 104, 162, 259, 600 ms) at each voxel position. No post-processing of the data was performed other than implemented by the vendor and .sdat/.spar files were used for further analysis. To quantify the final metabolites PRiOr knowledge FITting (ProFit, version 2.0) was used and adapted for water referencing^23^. The 2D spectrum of the water signal was fitted based on a simulated 2D water signal and using the same general model as for the metabolites (^23^, Equation 1) but without the model-free envelope. 1D water scans were fitted with LCModel and analyzed using in-house MATLAB code to derive correction factors for the relaxation attenuation of the 2D water signal. Segmentation was used to correct for the cerebrospinal fluid fraction in the measured voxels. With that we derived concentrations estimates in institutional units (IU) which are only roughly comparable to molal units (moles of metabolites per mass of brain water (excluding cerebrospinal fluid)), as we have not corrected for T1 or T2 relaxation attenuation of the metabolite signal. Cramer-Rao Lower Bounds percentage values resulting from ProFit fitting were used to assess spectral quality^23^.

Results of the MRS pipeline were then carefully visually inspected for abnormalities in modeled fits in the 1-D projection and the 2-D spectra by qualified personnel. Cases with artifacts caused by remaining lipid peaks or residual water peaks were rejected in order to ensure valid results of the statistical analyses. The visual inspection applied here resulted in the removal of seven cases, four in the tinnitus group and three in the control group. We did not perform any further exclusion based on other quality parameters (e.g., linewidth of the spectral fit or Relative Cramer-Rao Lower Bounds). Yet, we calculated or extracted these values alongside the ProFit estimation of the water peak area and present them in the Supplemental Material section for the interested reader (please note that these measures are solely presented for the final GABA subset after outlier removal). Moreover, we checked for differences in the voxel compositions between groups and hemispheres with respect to white matter, grey matter, and cerebrospinal fluid. This procedure ensures that the quantification of the metabolite spectra is feasible as well as valid with respect to underlying brain tissues and their inherent metabolite concentration distributions.

### Statistical analysis

For the participants’ characteristics we report means, standard deviations (*SD*), and median values. To test for differences in group matching we used chi-square tests for the categorical variable sex, whereas either *t*-tests or Wilcoxon rank sum tests were used for differences in numerical variables.

Acquired MRS metabolite concentrations for the two groups and voxels were analyzed using frequentist parametric statistics. Voxel composition differences between groups and hemispheres were checked with *t*-tests in an ancillary analysis to ensure feasibility of the actual analysis of metabolites. Following our rationale and hypotheses, GABA and Glu were analyzed separately. This included the identification and removal of outliers with the criterion of $\ge$ 2 standard deviations, which is akin to^16^. In order to test for differences in metabolite concentrations between groups and for interactions of group and voxels (i.e., hemispheres), distributions were first checked for normality with Shapiro-Wilk tests. The resulting normal distributions allowed for ANOVA tests for the main effects Group and Hemisphere as well as their interactions. Post hoc contrasts comparing metabolite concentrations per group and hemisphere were performed with Tukey Honest Significant Difference (HSD) tests. Cohen’s d was calculated to estimate the effect sizes of the group comparisons.

Exploratory correlation analyses were performed with the non-parametric Spearman method to account for non-normal distributions. Two-tailed tests were used due to the partly novel and exploratory nature of our contrasts and the overall *p*-value threshold for significance was set to $\le$ 0.05 (*) or to $\le$ 0.1 (${\dag }$) for trends. All statistical analyses were performed using RStudio IDE (RStudio: Integrated Development for R. RStudio, PBC, Boston, USA, http://www.rstudio.com/) integrating the R engine (R Foundation for Statistical Computing, Vienna, Austria) with vanilla statistical functions and the packages ggplot2^51^ or corrplot for plotting.

## Results

### Participant characteristics

Participants’ characteristics and group matching statistics are presented in Table 1. In the tinnitus group 4, of the 16 were female (vs. 4 of 17 in the control group; no difference between groups, *p* $=$ 0.99). The result of our questionnaire showed that of our 16 included tinnitus patients, two reported unilateral left sided tinnitus, three unilateral right sided tinnitus, and 11 bilateral tinnitus. All group matching criteria were met (*p*(min) $=$ 0.054 for LDL on the right ear, *p* $=$ 0.085 for hyperacusis (GUEF) score, all other *p* values > 0.1).

**Table 1:** Participant characteristics.

Variable	Tinnitus			Controls			Difference
	Mean	SD	Median	Mean	SD	Median	p
Age (years)	37.56	10.56	39	37.71	11.12	37.14	0.97
BAI (total score)	5.25	3.96	5.0	4.23	3.29	3	0.431
BDI (total score)	7.38	4.08	7.0	4.76	2.99	6	0.21
Hyperacusis (GUEF, total score)	7.19	6.36	6.0	3.82	4.13	3	0.085
HL left (dB)	7.66	3.69	8.0	8.12	3.48	8	0.714
HL right (dB)	9.56	2.99	9.5	9.0	3.74	9.0	0.636
LDL left (dB)	90.0	7.30	90	94.12	10.34	90	0.195
LDL right (dB)	87.5	7.53	85	93.82	10.39	90	0.054
PSQ (total score)	35.38	11.58	34	30.12	13.00	30	0.228
WHO (physical health)	86.19	6.88	86	84.70	9.48	86	0.61
WHO (psychological)	76.44	13.22	75	79.76	12.20	75	0.459
Tinnitus duration (months)	44.43	64.25	20				
MML left (dB)	25.36	17.59	22.5				
MML right (dB)	20.71	15.42	20				
RI level left (dB)	39.61	14.20	35				
RI level right (dB)	37.67	15.90	40				
RI rating left (NRS)	− 2.07	2.98	− 3				
RI rating right (NRS)	− 1.2	2.98	− 3				
THI (total score)	20.75	15.10	19				
Tinnitus frequency left (kHz)	10.39	4.70	10				
Tinnitus frequency right (kHz)	11.69	3.82	10.6				
Tinnitus loudness left (dB)	28.07	24.62	25				
Tinnitus loudness right (dB)	26.07	21.41	25				

*N* = 33 (16 tinnitus, 17 controls). All dB values are dB HL. Left and right refer to the two ears.

*SD* standard deviation, *NRS* numeric rating scale, *BAI* Beck Anxiety Inventory^37^, *BDI* Beck Depression Inventory^36^, *GUEF* Geräuscheüberempfindlichkeits-Fragebogen^41^, *HL* hearing loss, *LDL* loudness discomfort level, *PSQ* Perceived Stress Questionnaire^34,35^, *WHO* WHO (quality of life-BREF)^33^, *MML* minimal masking level, *RI* residual inhibition, *THI* Tinnitus Handicap Inventory^39,40^.

### Audiometry and tinnitometry

**Figure 2:**
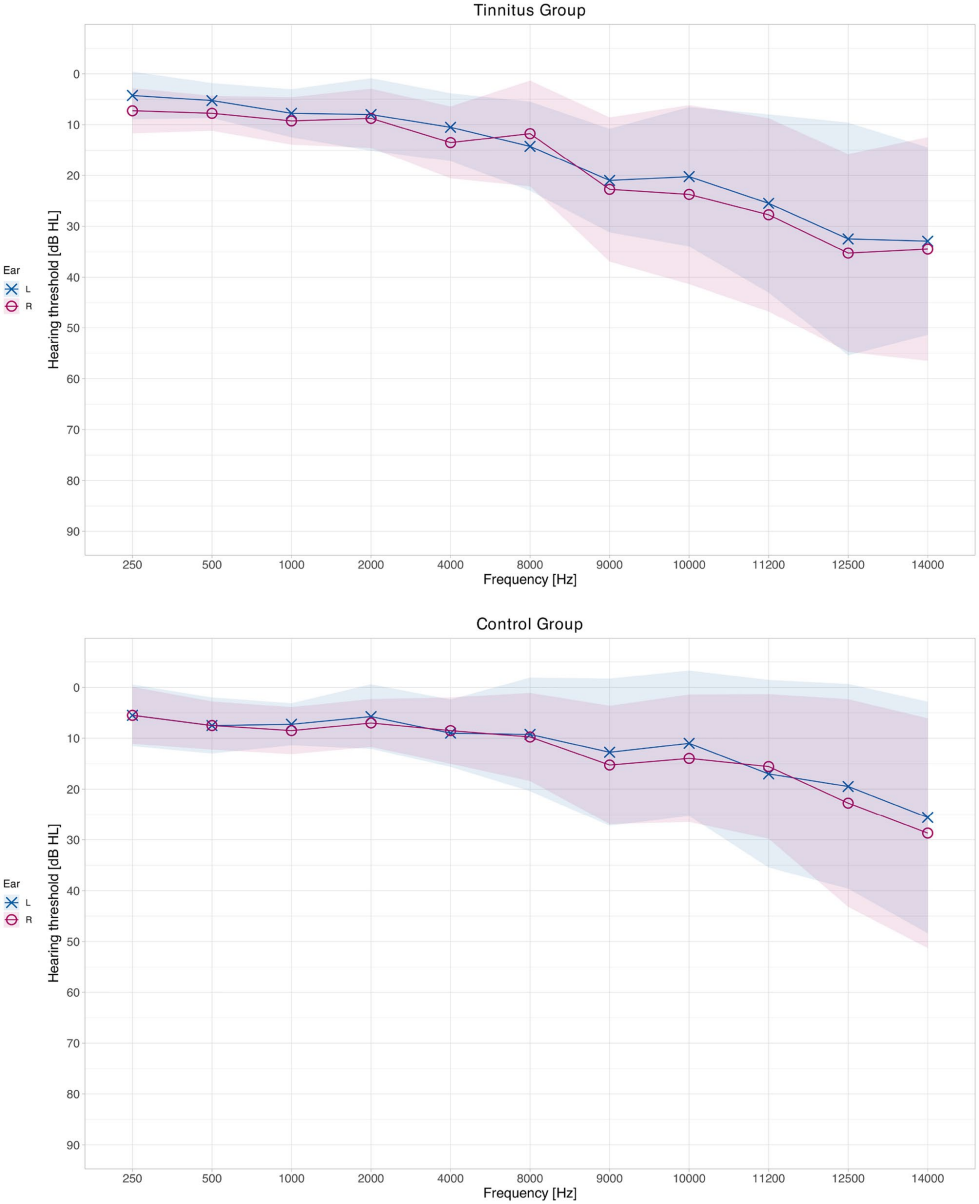
Hearing thresholds of both groups. The colored ribbons indicate standard deviations.

Audiometric results are plotted in Fig. 2 and indicated in Table 1 alongside the values of the tinnitometric tests.

### MRI

#### Morphometry

No anatomical differences were observed in the whole-brain analysis of cortical volume, thickness, and area (*p* > 0.05, for cortical surface thickness, area, and volume).

#### Spectroscopy: voxel composition and quality check

No differences were found in the two measurement voxels in left and right auditory cortex for both groups with regards to white matter, grey matter, and cerebrospinal fluid, respectively (ANOVAs, *p*(min) $=$ 0.21; Tukey HSD post hoc tests, *p*(min) $=$ 0.136). 5 and 2 outliers were removed according to our defined criterion (i.e., 2 SD in the metabolite concentration) over all groups and hemispheres for GABA and Glu, respectively. Cramer-Rao Lower Bounds and linewidth values for the GABA subset were in acceptable ranges for the critical metabolites and are listed in supplemental Table S1, supplemental Table S2, and supplemental Fig. S2. Furthermore, we also present ProFit water peak area estimates in supplemental Fig. S3. No differences were found in these parameters between groups.

#### Spectroscopy: GABA and glutamate

**Figure 3:**
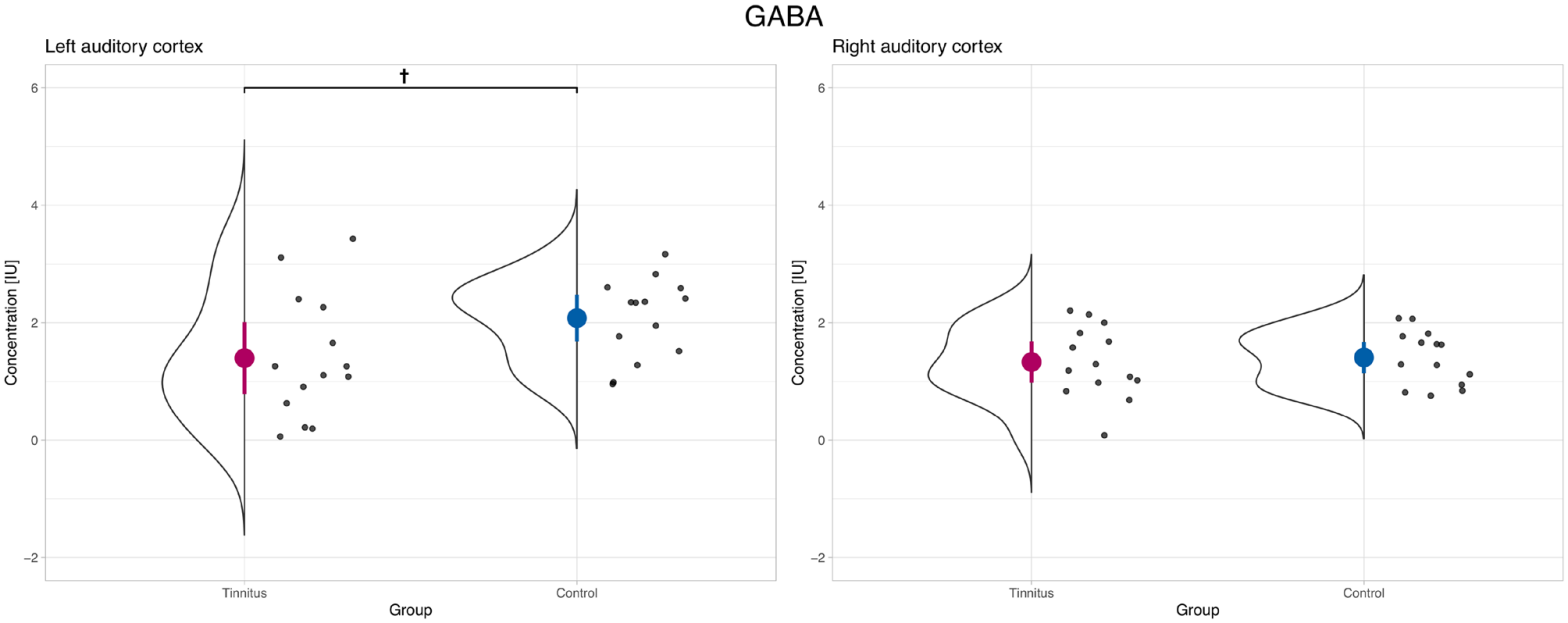
GABA concentration levels in left and right auditory cortex. The colored dot denotes the sample mean. The error bar covers the lower and upper Gaussian confidence limits based on the *t*-distribution. *IU* institutional unit.

**Figure 4:**
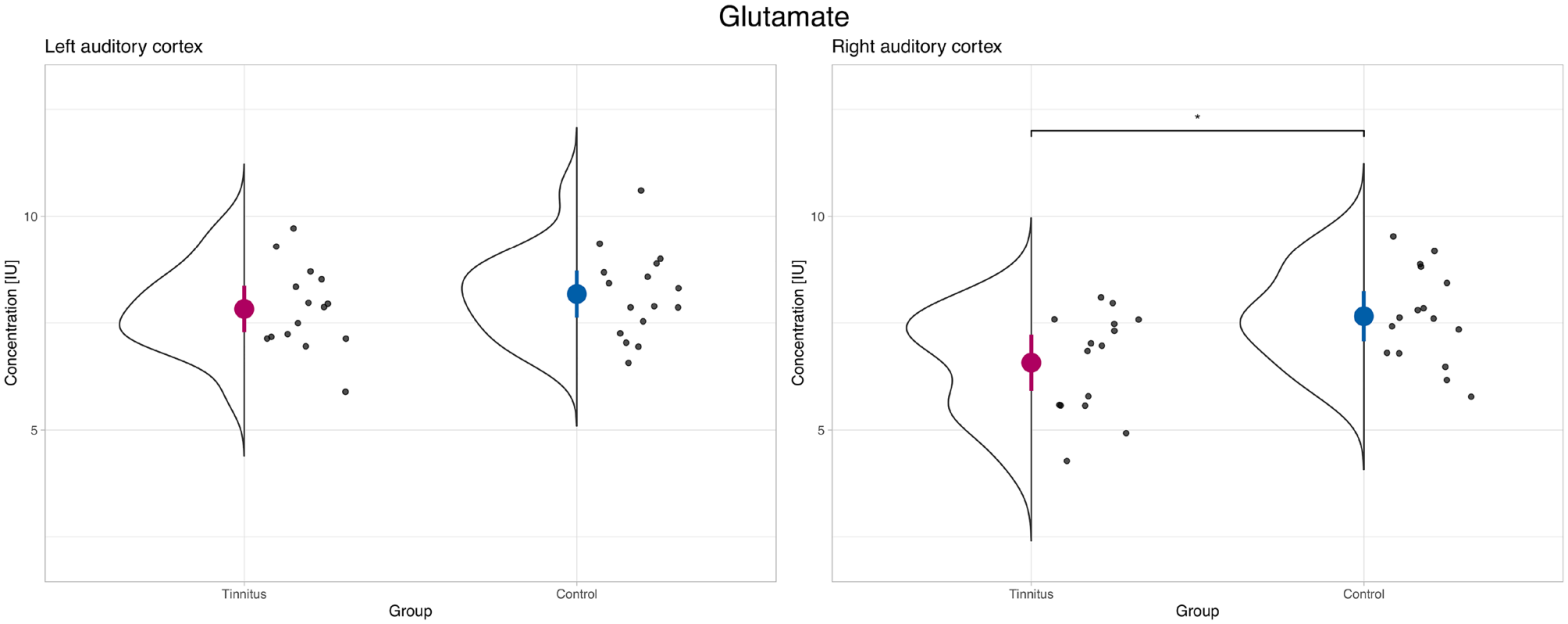
Glu concentration levels in left and right auditory cortex. The colored dot denotes the sample mean. The error bar covers the lower and upper Gaussian confidence limits based on the *t*-distribution. *IU* institutional unit.

**Figure 5:**
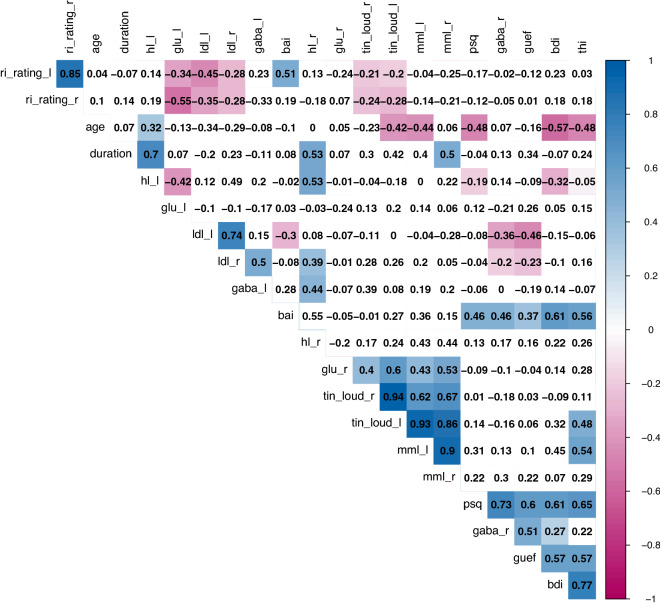
Correlation matrix of variables of interest and GABA/Glu concentration levels in the tinnitus group (*n* $=$ 16). Spearman correlations were used. Colored tiles indicate significant correlations (*p* < 0.05) and correlation strengths are coded in color saturation (positive $=$ blue, negative $=$ red). Correlation coefficients are automatically sorted with the built-in hierarchical clustering function of the corrplot package for better navigation of results ( *psq* Perceived Stress Questionnaire^34,35^, *gaba*_*r* GABA concentration in the right hemisphere ROI, *bai* Beck Anxiety Inventory^37^, *guef* Geräuscheüberempfindlichkeits-Fragebogen^41^, *bdi* Beck Depression Inventory^36^, *thi* Tinnitus Handicap Inventory^39,40^, *ri*_ *rating*_ *l* residual inhibition left ear, *ri*_ *rating*_ *r* residual inhibition right ear, *hl*_ *l* mean hearing loss left ear, *hl*_ *r* mean hearing loss right ear, *tin*_ *loud* _ *r* tinnitus loudness right ear, *tin*_ l*oud*_ *l* tinnitus loudness left ear, *mml*_ *l* minimal masking level left ear, *mml*_ *r* minimal masking level right ear, *gaba*_ *l* GABA concentration in the left hemisphere ROI, *ldl*_ *l* loudness discomfort level left ear, *ldl*_ *r* loudness discomfort level right ear).

The results of the ANOVAs and post hoc contrasts for GABA and Glu are listed in Table 2. Figures 3 and 4 show the metabolite concentration levels for the two groups and hemispheres. Figure 5 plots the correlation matrices for the metabolites and variables of interest.

The ANOVA model for GABA produced trends for the two factors (i.e., Group and Hemisphere). For Glu, significant effects were identified for Group and Hemisphere. In neither of the ANOVA models, significant or trend results were found for the interaction. A difference was found between groups for Glu in right auditory cortex in the post hoc contrasts indicating a reduced Glu concentration in the tinnitus group (*p* $=$ 0.034, Tukey HSD adjusted; *Cohens’d* $=$ 0.95, indicating a large effect). Regarding GABA, we can report a trend of reduced GABA concentration in the left auditory cortex (*p* $=$ 0.082, Tukey HSD adjusted; *Cohens’d* $=$ 0.76, indicating a medium effect). Following our secondary hypotheses, we can report positive correlations for bilateral tinnitus loudness and MML with Glu in the right hemisphere as well as negative correlations between bilateral RI and Glu in the left hemisphere (see Fig. 5). Finally, we present the results of 16 further metabolite concentrations in the supplemental Fig. S1 for exploratory purposes.

**Table 2:** ANOVA results for the models of GABA and Glu.

Anova			Post hoc *t*-tests Group*Hemisphere						
Factor	*F*	*p*	Hemisphere	Mean	Diff	Lower	Upper	*p* (adj.)	*Cohen’s d*
**GABA**									
Group	3.682	**0.061**†	Left	0.34	0.68	− 0.06	1.421	** 0.082**†	** 0.76**
Hemisphere	3.483	**0.068**†	Right	0.3	0.076	− 0.664	0.817	0.993	0.15
Group*Hemisphere	2.349	0.132							
**Glu**									
Group	6.862	0.011*	Left	1.99	0.35	− 0.675	1.375	0.803	0.35
Hemisphere	10.233	0.002**	Right	1.48	1.085	0.06	2.101	0.034*	** 0.95**
Group*Hemisphere	1.799	0.185							

Post hoc contrasts compare concentration levels between groups per hemisphere and are Tukey (HSD) adjusted. Confidence intervals are indicated by lower and upper bounds.

Significant values are in bold.

## Discussion

To date, no single model can be applied to explain all the phenomena and observations surrounding the perception of tinnitus and thus many paradoxes remain^52^. In the last decades, results from animal studies indicated that tinnitus may be part of a homeostatic dysregulation of the auditory cortex in response to auditory insults^53^. This dysregulation is mainly explained by altered neuronal excitability and imbalanced excitation and inhibition. In animal studies with tinnitus induced by acoustic trauma, neuronal inhibition has been shown to be reduced^6^. Following up on previous findings by^16^, in which an association between GABA and choline concentration in the auditory cortex with tinnitus could be established, we aimed to replicate and expand on previous results using a similar approach. GABA is an important neurotransmitter throughout the entire nervous system and the main inhibitory neurotransmitter in the central auditory system^12^. Glu counteracts GABA as an excitatory neurotransmitter in the entire auditory system from the cochlea up to the auditory cortex^22,54,55^. The neurons of the auditory cortex contain several types of receptors for GABA as well as different types of Glu receptors with various levels of affinity and influence on excitation or inhibition^21,56^. Moreover, Glu is the substrate for GABA, a particular which hampers the differentiation of metabolic vs. functional aspects of the respective neurotransmitter concentrations^22^. In order to further explore the role of GABA and Glu in tinnitus, we measured both concentrations in the primary auditory cortex of tinnitus patients and matched healthy controls. Given the constraints and general sensitivity of the MRS method, it was of utmost importance to produce precise and accurate measurements of GABA and Glu, and to differentiate between Glutamate and Glu by the application of a 2D-JPRESS MRS sequence with water reference. Furthermore, we expanded existing research findings in the addition of several auditory and psychometric measures, which we compared between our study groups and related to neurotransmitter concentrations by means of correlation analyses.

We hypothesized that GABA concentrations would be lower and Glu concentrations would be higher in the primary auditory cortex in our tinnitus group compared to a matched control group. Furthermore, we expected meaningful correlations of tinnitus loudness measures with GABA (negative correlation) and Glu (positive correlation) as well as tinnitus duration (negative correlation with GABA, positive correlation with Glu).

In our results we identified (1) an increased Glu concentration in the right hemisphere (large effect) of our control group compared to the tinnitus patients; (2) a reduced GABA concentration in the left hemisphere (medium effect) in the tinnitus group compared to the controls. The GABA concentration finding is in support of our hypothesis, and thus replicates the findings of^16^, while limited to the left auditory cortex in contrast to right auditory cortex in the former study. Our results are very similar in extent to the results of the former study, which is also reflected in the absence of significant interactions as well as trends on certain outcome variables while effect sizes are moderate to large in our study. In contrast, the increased Glu concentrations in the control group came as a surprise. Besides these differences in absolute concentration levels between study groups, we identified hypothesized correlations between Glu concentration levels and measures of tinnitus loudness (i.e., bilateral tinnitus loudness and MML with Glu in the right hemisphere) and negative correlations between Glu concentration levels and RI (i.e., bilateral RI with Glu in the left hemisphere), whereas no correlations were found for tinnitus perceptual parameters or duration with GABA.

As mentioned, our results with respect to Glu were unexpected and currently an elegant explanation within which to contextualize them remains unknown. In the following, however, we attempt to embed these findings within the current knowledge of Glu and tinnitus. Glu activates metabotropic and ionotropic receptors (mGluRs) which are theorized to play a key role in tinnitus^56^. These receptors for Glu are found along the entire auditory pathway including the auditory cortex^54^. MGluRs function via glial, pre-, and postsynaptic mechanisms (along with other receptors) as modulators of the excitability, and have been shown to negatively modulate excitatory neurotransmitter output. It has been hypothesized that these receptors have evolved to monitor Glu excess in the synapse and to prevent neuronal excitability^57^. In studies with mice it could be established that different groups of mGluRs altered neuronal firing and suppression^58^. Moreover, the phenomenon known as “residual inhibition” describes the short-term inhibiton of tinnitus sensation, after an appropriate masking sound^59^. While its underlying mechanisms are not fully explainable yet, a suppression of the spontaneous firing rate in the auditory pathway could play a critical role in the induction of residual inhibition. Based on the hypothesis that agonists on mGluR Group II would suppress the spontaneous firing in auditory neurons (as has been observed during residual inhibition tasks), a drug study showed tinnitus suppression due to the application of a Group II specific agonist^56^. In our experiment, we could demonstrate a lower concentration of Glu in the right auditory cortex of our tinnitus group compared with a normal hearing matched control group (see Fig. 4). In our view, the enhanced spontaneous firing rate after peripheral auditory injury in people suffering from tinnitus is not met with an adequate response of the metabotropic Glu receptors along the auditory pathway and thus the tinnitus sensation is not suppressed. In conclusion, we surmise that the observed decreased Glu concentration in the auditory cortex in the tinnitus group reflects this mismatch.

Further, we determined a correlation between experienced tinnitus loudness and the concentration of Glu in the right auditory cortex (see Fig. 5). This correlation is in line with the results of Cacace and colleagues^19^, in which transcranial magnetic stimulation of the left hemisphere auditory cortex led to a lower Glu concentration and a decrease in tinnitus loudness. In isolation, this result fits the reasoning about Glu being a marker for excitation and loudness in tinnitus although, in a broader context, the results did not reflect the expected difference in absolute concentration levels between groups. This observation may reflect a larger within-group (i.e., tinnitus) than between-group variability, a possibility which underscores the relevance of efforts to extend research within the tinnitus spectrum instead of solely relying on group contrasts with healthy controls^60,61^.

A well-established concept links the tinnitus sensation with increased spontaneous activity and reduction of inhibitory neurotransmission markers^7,8^. This deficit of inhibitory function is found throughout the auditory system including the dorsal cochlear nucleus, the inferior colliculus, and the primary auditory cortex^62,63^. A plausible cause of this change in homeostatic plasticity is the compensation of peripheral excitatory drive after peripheral hearing loss. These pre- and postsynaptic compensatory changes following deafferentation have been shown to cause effects on the GABAergic function in central target structures^64^. In the auditory cortex of rats it could be established that presynaptic glutamic acid decarboxylase levels were reduced and the postsynaptic GABA receptors altered due to age-related deafferentation^65,66^. A similar effect may take place in tinnitus generation, in which a peripheral auditory injury leads to decreased inhibitory neurotransmitter release and decreases in postsynaptic receptor density or type. In rats without hearing loss, tinnitus could be linked to an enzyme in axonal terminals:^21^ established a correlation between the severity of tinnitus in rats and the down-regulation of the expression of GAD65, a protein that influences the neuronal activity and the amount of GABA release. Although no clear-cut effects could be established in our analysis of GABA, we are confident that our results, showing a lower GABA concentration in the tinnitus group on the left hemisphere compared with a matched control group (*p* $=$ 0.081), are a valuable contribution to the ongoing discussion in line with previous findings and reasoning. In the right hemisphere, no difference between the groups could be found. This result contrasts with the findings reported by^16^, in which an effect was found in the right hemisphere and merely a trend in the left hemisphere. Notably, the investigated study populations differ with respect to laterality in that^16^ examined tinnitus patients with unilateral tinnitus whereas we concentrated on bilateral tinnitus. As mentioned above, our group of tinnitus patients included all tinnitus variants with a majority experiencing bilateral tinnitus. In any case, while details of laterality of tinnitus may play a role and certainly have to be considered in future research, our findings of potentially lowered GABA concentrations in auditory cortex of tinnitus patients is in line with former findings and theoretical reasoning. In contrast to the analysis of Glu, we could not find any meaningful correlation of GABA with tinnitus or audiometric parameters, which is in line with the former study of^16^. Unfortunately, given the absence of fitting contextual references, we can only speculate about the reasons, such as methodological aspects particular to MRS (e.g., voxel size and coverage) or characteristics of the study population (here: healthy, middle-aged subjects with no hearing loss compared to studies in presbycusis^13,29^).

The differences in GABA concentrations are less pronounced than in the study of^16^ which could be related to the recently reported lower test-retest reliability for 2D-JPRESS compared to MEGA PRESS^26^. Yet, our coefficients of variance from Glu and GABA in the critical left voxel in our control group are lower than in the former methodological comparison study (coefficient of variance of glutamate-glutamine complex right $=$ 15%, left $=$ 13%; coefficient of variance of GABA right $=$ 56%, left $=$ 39%). We surmise that given these partly conflicting results the question of superiority of MRS protocols remains to be elucidated in further studies.

GABA and Glu are omnipresent neurotransmitters in the central nervous system and part of many basic metabolic processes like the citric acid cycle^67^. It could therefore be that measured concentration levels are confounded or masked by these basic metabolic processes and in consequence the signal-to-noise ratio of our analysis might be lowered with respect to the targeted pathological differences in concentrations. Given that these basal metabolic processes are putatively present in both groups of this study and no indications exist that these could play a role in tinnitus pathophysiology, we surmise that this possible confounder can be ruled out in the presented results and the found differences between groups represent true effects. Furthermore, the present method is not capable of discerning synaptic alterations from altered neurotransmitter levels pertinent to tinnitus pathophysiology. In human neuroscience, hearing-related and tinnitus-related synaptopathy may currently only be studied with non-invasive electrophysiology^68^. Therefore we are not able to further elucidate the causality or direction behind observed neurotransmitter alterations and reported synaptopathy in tinnitus. Looking at our studied population with no hearing loss pathological synaptic alterations may also be absent or merely minimally present. It is currently impossible to study the metabolism of (human) primary auditory cortex in vivo in more spatial detail and thus accessing neurons or neuronal populations specifically involved in tinnitus pathophysiology. With the current method and data, we are here limited to cautiously interpret the observed differences as possible changes of any feature (e.g., synaptopathy) of underlying tinnitus-specific neuronal populations.

MRS offers a wide range of possibilities to quantify concentrations of molecules like neurotransmitters. Using the 2D-JPRESS method to identify small concentrations of neurotransmitters like GABA, the measurement with a 3 T MRI requires a certain size of ROI in order to achieve a sufficient signal-to-noise ratio at each measured echo time and to acquire reliable data. Thus we were forced to define a ROI that included other areas adjacent to the primary auditory cortex. Large voxel sizes are also required for editing sequences like MEGA PRESS. Another disadvantage of the large voxel size is the poorer shim quality. In addition, the proximity to the extra-cranial lipids led to the relatively numerous measurements that had to be excluded due to artifacts. Finally, overall signal quality could have been improved by counterbalanced voxel acquisition (i.e., randomization of scanning order between right and left hemisphere in our study.) Yet, given the quality checks performed and presented in this study, we surmise that the influence of the missing counterbalancing is negligible. Based on the visually-inspected signal-to-noise ratio achieved in this work, it should be possible for subsequent studies to reduce the voxel size at this position in the brain for improved shim quality without major consequences for the 2D-JPRESS evaluation. Alternatively, we recommend the use of 7 T scanners to further constrain the measurement to a ROI containing the auditory cortex only. Despite all our quality checks and attempts to control possible confounders, we report GABA values, especially in the tinnitus group and the left hemisphere, which are close to 0 and thus prima facie physiologically implausible. In the absence of any critical issue with signal quality, we conclude that the observed near-zero values are most probably an unfortunate interaction between a true effect and current limitations of the MRS method. Of special note, the former study of Sedley and colleagues^16^ also reported a single outlier near zero using the MRS PRESS sequence. Further, it would have been advantageous to include a third ROI as a reference region independent of the auditory system. However, our current scan procedures are already very tedious for the participants and any further scanning time would have put even more strain on them. Moreover, scanner noise is a considerable burden for tinnitus patients in general and thus certainly further limits the extent of acceptable scanning time. The MRS sequence applied in this study produces knocking sounds in low frequencies of the audible spectrum which are less disturbing than other louder MRI sequences. Participants were sufficiently protected against scanner noise and did not report any changes in tinnitus or other adverse events. We experienced difficulty in finding participants with a high tinnitus burden, reflected by high THI scores, as well as fitting the other matching criteria, such as age and absence of hearing loss, in our locally accessible study population. The quantification of concentrations of the neurotransmitters of interest resulted in different levels in each hemisphere. As our group included mostly both-sided tinnitus, in contrast to the unilateral comparison of^16^, we cannot rule out any influences of tinnitus lateralization in our data. Future studies may therefore include a more comprehensive, large sample, which covers the whole spectrum of tinnitus lateralization thus allowing respective statistical contrasts. On the other hand, single-sided tinnitus in the absence of hearing loss is rare. Given that we designed our study to investigate tinnitus without hearing loss, we focused on a sample with bilateral tinnitus, a sample which can also be considered more representative of the general tinnitus population. Tinnitus matching may be extended and improved in future studies to ensure validity of matched tinnitus pitch and loudness. Loudness should ideally be matched to a reference tone to avoid confusions or interactions between matched frequency and loudness^69,70^. Due to the nature of this study, it is not possible to conclude whether our findings of GABA and Glu are cause or consequence of the tinnitus sensation. Longitudinal or (pharmacological) intervention studies would shed more light on the issue of causation, the time course, or reversibility of such changes or differences in neurotransmitter concentrations.

In conclusion, our study could demonstrate reduced Glu concentration levels in right auditory cortex and a reduction of GABA in the left auditory cortex of tinnitus patients with no significant hearing loss compared to matched healthy controls. These results are novel given that it was the first study to quantify cortical Glu concentrations in a group contrast in human subjects (i.e., tinnitus patients vs. healthy controls). Reduced GABA levels are in line with former findings and theoretical reasoning and may reflect dysfunctional inhibition/excitation patterns in auditory cortex of individuals with tinnitus. The finding of reduced Glu levels, on the other hand, came as a surprise and can currently not be conclusively interpreted with respect to tinnitus. In normal brain metabolism, Glu is the precursor of GABA which, while serving as a possible explanation here, complicates the interpretation of findings from a previous treatment study as well as our hypothesized observation of a positive relation between Glu levels and tinnitus loudness. Alternatively, we reason that the reduced Glu levels may be dependent on dysfunctional suppression of spontaneous hyperactivity in the auditory pathway after peripheral auditory injury mediated by mGluR receptors. Future studies would profit from the addition of further tinnitus subgroups to allow for a more in-depth study of tinnitus parameters. Longitudinal studies could furthermore shed more light on interactions of brain metabolism and chronification of tinnitus. MRS studies in tinnitus are deemed highly valuable with respect to modeling, causality, diagnostics, and interventions.

## Supplementary Information

Supplementary Information.

## Data Availability

The authors confirm that the data supporting the findings of this study are available within the article and/or its Supplementary Material. Data can be made available upon reasonable request.
