# Supplementary material for: Lower glutamate and GABA levels in auditory cortex of tinnitus patients: a 2D-JPRESS MR spectroscopy study
Source: Sci Rep. 2022 Mar 8;12:4068. doi: 10.1038/s41598-022-07835-8 (PMC8904839; doi:10.1038/s41598-022-07835-8)
Supplement: Supplementary file 1 — Supplementary Information. [file 41598_2022_7835_MOESM1_ESM.pdf]

Supplemental Material

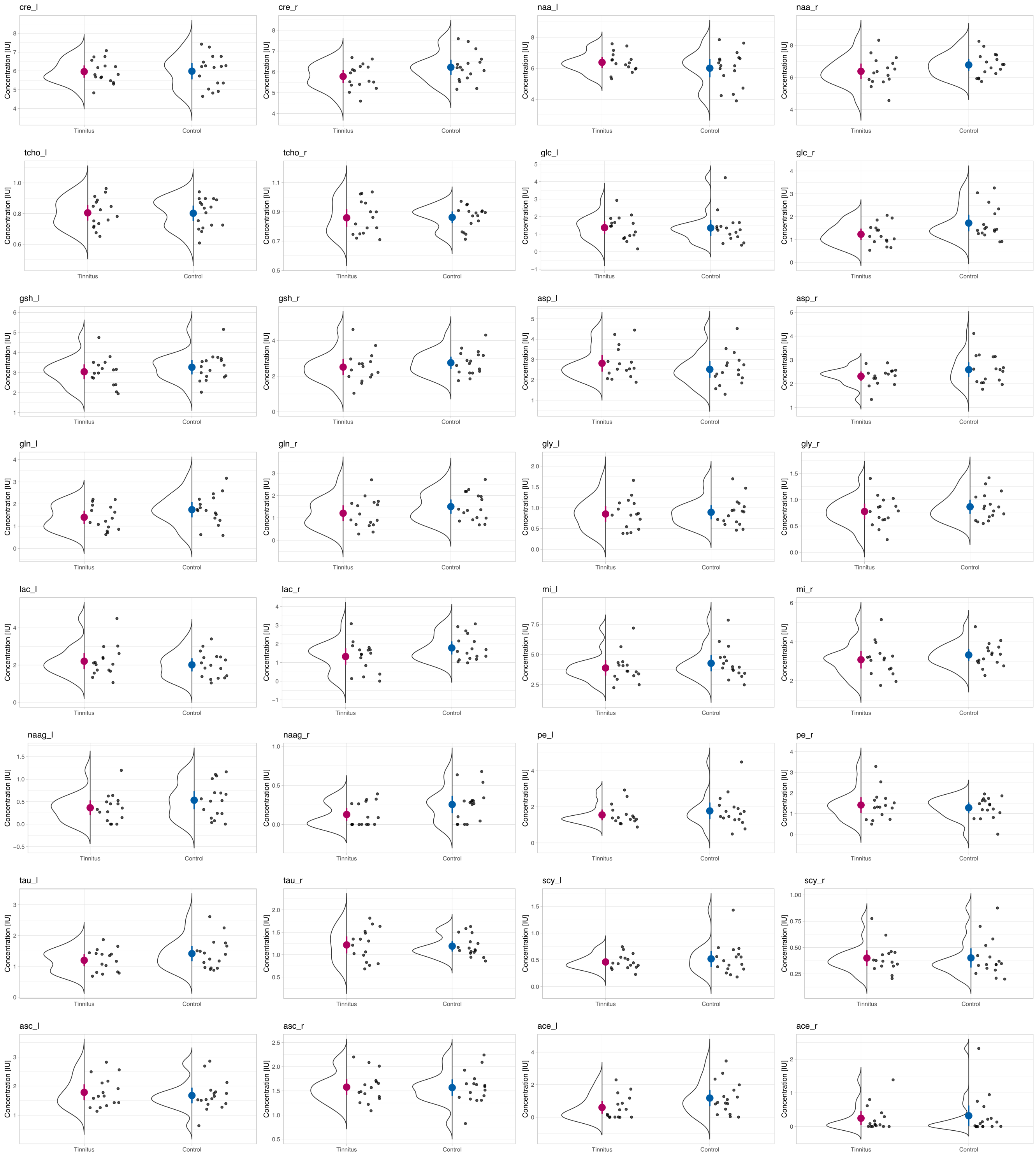

Figure S1. Metabolite concentration levels in left and right auditory cortex. The colored dot denotes the sample mean. The error bar covers the lower and upper Gaussian confidence limits based on the t-distribution. No outlier removal was performed. No differences were found in an exploratory analysis testing for differences in the two hemispheres between groups ($p > 0.05$) except for glucose in right auditory cortex ($p = 0.025$). l = left. r = right. cre = Creatine. naa = N-Acetylaspartic acid. tcho = Choline. glc = Glucose. gsh = Glutathion. asp = Aspartate. gln = Glutamine. gly = Glycine. lac = Lactate. mi = MyoInositol. naag = NAcetyl Aspartyl Glutamate. pe = Phosphorylethanolamine. tau = Taurin. scy = Scylloinositol. asc = Ascorbic acid. ace = Acetate. IU = Institutional Unit.

	Tinnitus					Control					Difference
Metabolite	Mean	SD	Median	Min	Max	Mean	SD	Median	Min	Max	p (t-test)
cre_l	2.49E-01	4.99E-02	2.34E-01	1.93E-01	3.63E-01	2.79E-01	7.84E-02	2.51E-01	2.02E-01	4.97E-01	0.250
naa_l	1.84E-01	3.42E-02	1.73E-01	1.41E-01	2.65E-01	2.17E-01	6.81E-02	1.94E-01	1.41E-01	4.09E-01	0.124
tcho_l	3.07E-01	6.57E-02	2.85E-01	2.49E-01	4.58E-01	3.56E-01	1.23E-01	3.12E-01	2.53E-01	7.28E-01	0.204
glc_l	1.45E+01	1.81E+01	7.26E+00	5.05E+00	7.44E+01	1.17E+01	6.73E+00	8.33E+00	4.68E+00	2.58E+01	0.601
gsh_l	1.74E+00	3.90E-01	1.66E+00	1.33E+00	2.67E+00	1.95E+00	6.49E-01	1.74E+00	1.29E+00	3.44E+00	0.325
asp_l	4.51E+00	1.58E+00	4.14E+00	2.96E+00	9.56E+00	4.91E+00	1.65E+00	4.63E+00	2.67E+00	8.53E+00	0.520
gaba_l	2.13E+01	2.82E+01	8.51E+00	3.69E+00	9.86E+01	7.39E+00	4.84E+00	5.25E+00	4.08E+00	1.82E+01	0.092
gln_l	9.01E+00	3.19E+00	8.19E+00	5.70E+00	1.75E+01	9.35E+00	6.49E+00	7.69E+00	4.82E+00	3.05E+01	0.862
glu_l	8.60E-01	1.81E-01	8.22E-01	6.66E-01	1.33E+00	1.03E+00	3.00E-01	9.05E-01	6.87E-01	1.75E+00	0.087
gly_l	7.77E+00	2.44E+00	7.05E+00	5.24E+00	1.27E+01	8.14E+00	2.39E+00	7.55E+00	5.06E+00	1.31E+01	0.688
lac_l	2.84E+00	8.81E-01	2.74E+00	1.51E+00	4.87E+00	2.70E+00	7.78E-01	2.48E+00	1.80E+00	4.67E+00	0.661
mi_l	9.68E-01	2.29E-01	8.91E-01	7.65E-01	1.52E+00	1.08E+00	3.86E-01	9.61E-01	7.52E-01	2.23E+00	0.374
naag_l	1.22E+12	2.80E+12	5.65E+00	2.03E+00	9.33E+12	5.12E+11	1.92E+12	4.24E+00	1.49E+00	7.17E+12	0.445
pe_l	6.87E+00	1.98E+00	6.17E+00	4.42E+00	1.02E+01	9.22E+00	4.74E+00	7.50E+00	4.39E+00	2.34E+01	0.105
tau_l	6.85E+00	3.96E+00	5.25E+00	4.22E+00	1.91E+01	6.76E+00	2.23E+00	6.42E+00	3.07E+00	1.19E+01	0.944
scy_l	2.48E+00	1.01E+00	2.29E+00	1.39E+00	5.67E+00	2.99E+00	1.34E+00	2.93E+00	8.24E-01	5.87E+00	0.268
asc_l	4.08E+00	1.02E+00	3.86E+00	2.83E+00	6.13E+00	4.83E+00	1.31E+00	4.23E+00	3.42E+00	7.63E+00	0.106
ace_l	4.96E+12	7.00E+12	1.82E+01	1.76E+00	1.94E+13	9.15E+11	3.42E+12	4.31E+00	1.44E+00	1.28E+13	0.067
cre_r	2.60E-01	3.36E-02	2.53E-01	2.09E-01	3.21E-01	2.45E-01	3.08E-02	2.36E-01	2.10E-01	3.00E-01	0.247
naa_r	1.90E-01	2.77E-02	1.86E-01	1.55E-01	2.44E-01	1.91E-01	2.79E-02	1.90E-01	1.33E-01	2.33E-01	0.963
tcho_r	3.01E-01	3.69E-02	2.92E-01	2.51E-01	3.71E-01	3.01E-01	4.65E-02	2.92E-01	2.53E-01	4.05E-01	0.969
glc_r	8.47E+00	3.39E+00	7.78E+00	4.18E+00	1.64E+01	7.17E+00	1.93E+00	7.02E+00	4.80E+00	1.13E+01	0.227
gsh_r	1.88E+00	3.44E-01	1.80E+00	1.34E+00	2.71E+00	1.82E+00	5.20E-01	1.65E+00	1.24E+00	3.30E+00	0.729
asp_r	4.04E+00	8.49E-01	3.72E+00	3.01E+00	6.18E+00	3.84E+00	7.04E-01	3.66E+00	2.83E+00	5.36E+00	0.488
gaba_r	1.14E+01	1.69E+01	6.13E+00	3.82E+00	6.87E+01	7.08E+00	1.85E+00	7.06E+00	4.88E+00	1.24E+01	0.358
gln_r	9.79E+00	3.92E+00	9.73E+00	3.36E+00	1.76E+01	9.24E+00	2.99E+00	9.22E+00	4.39E+00	1.48E+01	0.680
glu_r	8.51E-01	1.19E-01	8.51E-01	6.55E-01	1.09E+00	8.36E-01	1.23E-01	8.15E-01	6.50E-01	1.10E+00	0.747
gly_r	7.60E+00	2.14E+00	7.20E+00	5.18E+00	1.25E+01	6.54E+00	1.13E+00	6.86E+00	3.74E+00	8.37E+00	0.117
lac_r	5.08E+11	1.90E+12	4.97E+00	2.71E+00	7.11E+12	4.40E+00	1.84E+00	3.58E+00	2.54E+00	7.70E+00	0.336
mi_r	1.04E+00	1.44E-01	1.01E+00	8.19E-01	1.32E+00	1.00E+00	1.67E-01	9.55E-01	7.65E-01	1.29E+00	0.461
naag_r	4.09E+12	4.30E+12	4.38E+12	3.98E+00	1.37E+13	1.87E+12	3.95E+12	7.10E+00	1.98E+00	1.27E+13	0.165
pe_r	9.67E+00	3.69E+00	8.30E+00	5.45E+00	1.78E+01	2.68E+12	1.00E+13	8.13E+00	4.24E+00	3.76E+13	0.336
tau_r	5.82E+00	1.02E+00	6.04E+00	3.85E+00	7.23E+00	6.32E+00	1.57E+00	5.82E+00	4.30E+00	9.80E+00	0.333
scy_r	2.68E+00	7.42E-01	2.56E+00	1.27E+00	4.21E+00	3.00E+00	1.06E+00	3.01E+00	9.08E-01	4.91E+00	0.357
asc_r	3.87E+00	9.70E-01	3.80E+00	2.61E+00	5.23E+00	4.01E+00	7.29E-01	3.87E+00	3.00E+00	5.21E+00	0.673
ace_r	2.44E+12	3.74E+12	4.32E+01	3.64E+00	9.44E+12	2.64E+12	3.71E+12	3.58E+01	5.56E+00	8.45E+12	0.887

Table S1. Relative Cramer-Rao Lower Bounds (CRLB) percentage values for all metabolites. Data presented here is the GABA subset (i.e., GABA outliers removed). No differences were observed between groups (p(min)=0.067 for ace_l). The median is a better parameter for central tendency given some singular large outliers in the relative CRLB values. Non-parametric testing did not qualitatively change the results (p(min)=0.062 for ace_l). SD = Standard deviation. l = left. r = right. cre = Creatine. naa = N-Acetylaspartic acid. tcho = Choline. glc = Glucose. gsh = Glutathion. asp = Aspartate. gln = Glutamine. gly = Glycine. lac = Lactate. mi = MyoInositol. naag = NAcetyl Aspartyl Glutamate. pe = Phosphorylethanolamine. tau = Taurin. scy = Scylloinositol. asc = Ascorbic acid. ace = Acetate.

	Tinnitus					Control					Difference
Linewidth FWHM [Hz]	Mean	SD	Median	Min	Max	Mean	SD	Median	Min	Max	p (t-test)
Left voxel	10.046	1.404	10.011	7.325	12.697	11.162	1.924	10.255	8.302	14.162	0.092
Right voxel	9.523	1.884	9.767	6.837	12.697	9.209	1.419	8.79	6.837	11.72	0.623

Table S2. Linewidths in the two voxels. Data presented here is the GABA subset (i.e., GABA outliers removed). No differences between groups were detected (left: t = -1.754, p = 0.092; right: t = 0.499, p = 0.623). FWHM = full width at half maximum.

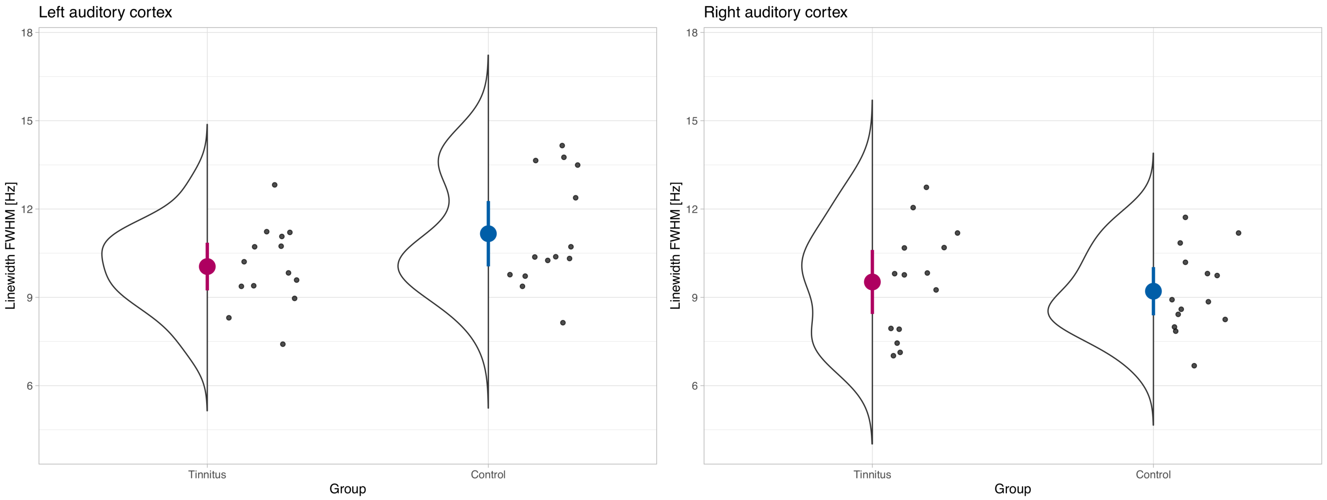

Figure S2. **Linewidths in the two voxels.** Data presented here is the GABA subset (i.e., GABA outliers removed). The colored dot denotes the sample mean. The error bar covers the lower and upper Gaussian confidence limits based on the t-distribution. See table S2 for descriptive and difference statistics. FWHM = full width at half maximum.

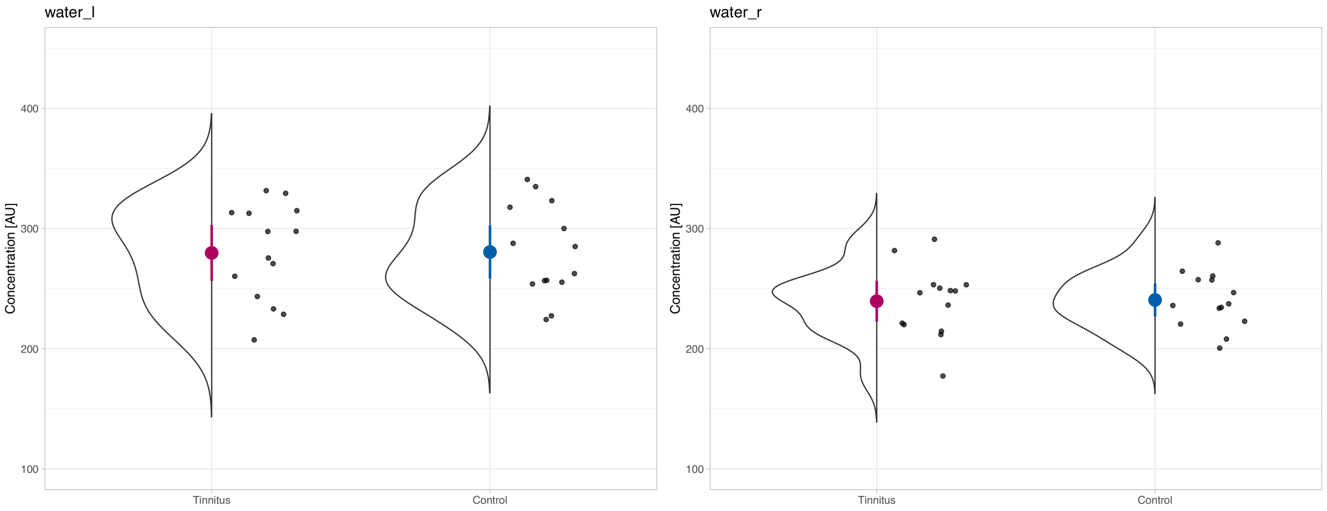

Figure S3. **ProFit water peak area estimation in the two voxels.** Data presented here is the GABA subset (i.e., GABA outliers removed). No differences between groups were detected (left: $t = -0.255$, $p = 0.8$; right: $t = -0.472$, $p = 0.64$). AU = arbitrary units. l = left. r = right.
